# Supplementary material for: Folding and Binding Mechanisms of the SH2 Domain from Crkl
Source: Biomolecules. 2022 Jul 22;12(8):1014. doi: 10.3390/biom12081014 (PMC9332313; doi:10.3390/biom12081014)
Supplement: Supplementary file 1 [file biomolecules-12-01014-s001.zip › biomolecules-1806557-supplementary.pdf]

Table S1 Kinetic (un)folding parameters of the Crkl SH2 domain calculated at different Na_2SO_4 concentrations.

$[Na_2SO_4]$ (M)	k_{IN} (s^{-1})	k_{NI} (s^{-1})	K_{IU}
0	26 ± 2	0.0084 ± 0.0007	0.0052 ± 0.0011
0.15	124 ± 9	0.0030 ± 0.0003	0.0013 ± 0.0003
0.3	150 ± 12	0.0015 ± 0.0001	0.0003 ± 0.0001

Note: The entire data set was globally fitted with Eq.2, sharing m_{I-N}, m_{N-I} and m_{I-D} values: m_{I-N} = 0.28 ± 0.05 Kcal mol^{-1} M^{-1}, m_{N-I} = 1.00 ± 0.01 Kcal mol^{-1} M^{-1}, m_{I-D} = 2.60 ± 0.05 Kcal mol^{-1} M^{-1}. The global m_{D-N} value was 3.9 ± 0.7 Kcal mol^{-1} M^{-1} calculated as the sum of m_{I-N}, m_{N-I}, and m_{I-D}.

Table S2. Kinetic (un)folding parameters of the Crkl SH2 domain calculated at different pH conditions in presence of 0.15 M Na_2SO_4.

**pH**	k_{IN} **(s^{-1})**	k_{NI} **(s^{-1})**	K_{ID}	m_{N-I} **(Kcal mol^{-1} M^{-1})**	m_{D-N} **(Kcal mol^{-1} M^{-1})**
4.0	9 ± 5	1.1490 ± 0.1543	0.2776 ± 0.2969	1.35 ± 0.04	4.13 ± 0.36
4.5	15 ± 1	0.0927 ± 0.0106	0.0428 ± 0.0077	1.36 ± 0.03	4.14 ± 0.33
5.0	35 ± 5	0.0192 ± 0.0019	0.0409 ± 0.0090	1.22 ± 0.02	4.00 ± 0.31
5.5	126 ± 17	0.0049 ± 0.0004	0.0485 ± 0.0107	1.12 ± 0.01	3.91 ± 0.30
6.7	122 ± 11	0.0040 ± 0.0005	0.0158 ± 0.0029	0.98 ± 0.02	3.76 ± 0.29
7.2	93 ± 8	0.0036 ± 0.0005	0.0014 ± 0.0002	0.98 ± 0.02	3.76 ± 0.29
8.0	55 ± 5	0.0034 ± 0.0005	0.0009 ± 0.0002	1.02 ± 0.02	3.80 ± 0.30
8.5	57 ± 5	0.0059 ± 0.0009	0.0010 ± 0.0002	0.97 ± 0.02	3.75 ± 0.29
9.0	63 ± 6	0.0110 ± 0.0016	0.0013 ± 0.0002	0.92 ± 0.02	3.70 ± 0.29

Note: The entire data set at different pH was globally fitted with Eq.2, sharing m_{I-N} and m_{I-D} values; m_{I-N} = 0.06 ± 0.06 Kcal mol^{-1} M^{-1}; m_{I-D} = 2.72 ± 0.05 Kcal mol^{-1} M^{-1}; m_{D-N} value = m_{I-N} + m_{N-I} + m_{I-D}.

Table S3 Kinetics parameters obtained from pseudo-first order binding reaction between the wild-type Crkl SH2 domain and $Pax_{112-123}$ peptide, at different ionic strengths and 283K.

[NaCl] (M)	k_{on} (μM^{-1} s^{-1})	k_{off} (s^{-1})	K_D (μM)
0.15	45.9 ± 2.2	20.4 ± 1.5	0.4 ± 0.3
0.3	24.7 ± 2.8	21.8 ± 1.1	0.9 ± 0.7
0.5	10.4 ± 1.4	28. 6 ± 0.5	2.8 ± 0.4
1	6.9 ± 0.6	39.6 ± 4.0	5.8 ± 0.7
